# Supplementary material for: Aquilariae Lignum Methylene Chloride Fraction Attenuates IL-1β-Driven Neuroinflammation in BV2 Microglial Cells
Source: Int J Mol Sci. 2020 Jul 30;21(15):5465. doi: 10.3390/ijms21155465 (PMC7432889; doi:10.3390/ijms21155465)
Supplement: Supplementary file 1 [file ijms-21-05465-s001.pdf]

Supplementary information

Materials and Methods

Cell Culture and Cell Viability

Murine hippocampal neuronal cells (HT22) was cultured in DMEM supplemented with 10% FBS and 1% penicillin-streptomycin, and cells was incubated at 37 °C under 5% CO_2. To evaluate cytotoxicity of ALF, the HT22 cells (4×10^3 cells/well) was seeded into 96-well microplates, and incubated for 12 h. Then, the cells were pretreated with ALF (0.5, 1, 2.5 μg/mL) or positive control (NAC, 100 μM) for 24 h. Meanwhile, the HT22 cells were pretreated with ALF (0.5, 1, 2.5 μg/mL) or positive control (NAC, 100 μM) for 2 h, and then cells were exposed to glutamate (20 mM) for 24 h in order to evaluate protective effects of ALF against neuroexcitotoxicity. Cell viability was evaluated with a WST-8 assay (EZ-Cytox, DoGen, Korea). Absorbance at 450 nm was measured using a UV spectrophotometer (Molecular Devices, CA, USA).

Results

Effects on the Cytotoxicity and Neuroexcitotoxicity in HT22 Cells

Cell viability was not altered by ALF (0.5, 1, 2.5 μg/mL) (Figure. S1A). Glutamate treatment significantly reduced cell viability compared with vehicle-treated cells ($p < 0.01$), whereas pretreatment with ALF significantly prevented neuroexcitotoxicity ($p < 0.01$ for 2.5 μg/mL, Figure. S1B). NAC similarly prevented also.

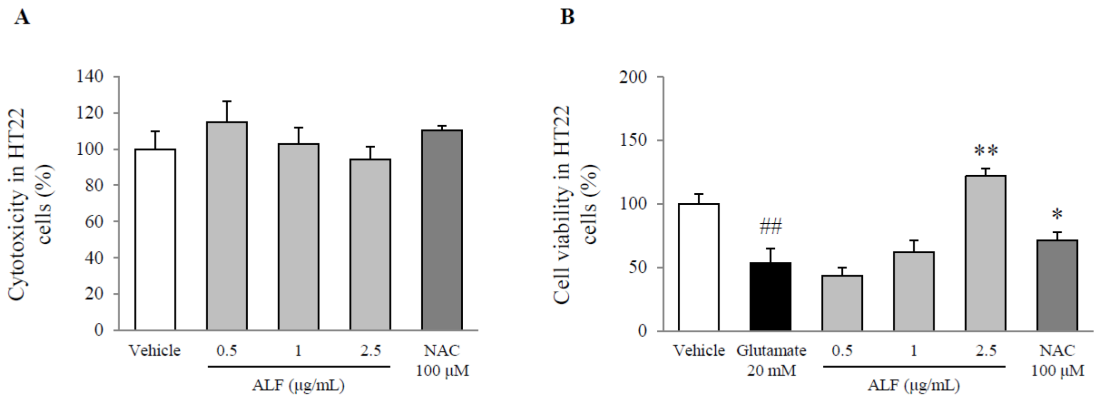

Figure S1. Cell viability. HT22 cells were pretreated with ALF for 24 h, and then cell viability was determined (**A**). HT22 cells were pretreated with ALF for 2 h before exposure to glutamate (20 mM) for 24 h, and then cell viability was determined (**B**). The data are expressed as the mean ± SD (n = 6). ##$p < 0.01$ compared with the vehicle-treated cells; *$p < 0.05$ and **$p < 0.01$ compared with the glutamate-exposed HT22 cells.
